# Curvature induced quantum phase transitions in an electron-hole system

**DOI:** 10.1038/s41598-018-34903-9

**Published:** 2018-11-07

**Authors:** Zhuo Bin Siu, Jian-Yuan Chang, Seng Ghee Tan, Mansoor B. A. Jalil, Ching-Ray Chang

**Affiliations:** 1 Department of Electrical and Computer Engineering, National University of Singapore, Singapore, Singapore; 2 Graduate Institute of Applied Physics and Department of Physics, National Taiwan University, Taipei, 10617 Taiwan; 3 Department of Physics, National Taiwan University, Taipei, 10617 Taiwan; 4 Department of Optoelectric Physics, Chinese Culture University, Taipei, 11114 Taiwan; 5 Center for Theoretical Physics, National Taiwan University, Taipei, 10617 Taiwan

## Abstract

In this work, we study the effect of introducing a periodic curvature on nanostructures, and demonstrate that the curvature can lead to a transition from a topologically trivial state to a non-trivial state. We first present the Hamiltonian for an arbitrarily curved nanostructure, and introduce a numerical scheme for calculating the bandstructure of a periodically curved nanostructure. Using this scheme, we calculate the bandstructure for a sinusoidally curved two-dimensional electron gas. We show that the curvature can lead to a partner switching reminiscent of a topological phase transition at the time reversal invariant momenta. We then study the Bernevig-Hughes-Zhang (BHZ) Hamiltonian for a two-dimensional quantum well. We show that introducing a curvature can lead to the emergence of topological surface states.

## Introduction

Quantum cosmology is intimately linked to space-time curvature^1–3^. It is however difficult to obtain experimental evidence of the curvature effect in the universe. The rapid progress in the fabrication of curved nanostructures can provide an experimental study of the curvature effect. More specifically, there is much interest in the investigation of spin-orbit coupling (SOC) and spin transport in curved two-dimensional (2D) systems^4–19^.

Quantum phase transitions, which have attracted a great deal of attention recently^20–25^, are phase transitions between different quantum phases at zero temperature. In particular, quantum phase transitions can be driven by tuning a non-temperature parameter^20^. Nevertheless, until now no study has yet investigated the curvature effect on quantum phase transitions. Recent theoretical and experimental studies both indicate that curvature is of considerable significance in curved nanostructures^4–19^. Hence, in a large-curvature system, it is necessary to fully understand the curvature effect on the quantum phase transition. The aim of this paper is to theoretically demonstrate the existence of the curvature-dependent quantum phase transition. Hence, in a large-curvature system, it is necessary to fully understand the curvature effect on the quantum phase transition.

In Section (II) we use the example of a two-dimensional spin (*σ*) system to illustrate how the Hamiltonian is modified in the presence of curvature. Here, spin (*σ*) refers to a generalized spin-like internal degree of freedom of the wavefunction over the SU(2) Bloch sphere. In Section (III), using the modified Hamiltonian, we numerically calculate the band-structure for the exemplary case of a periodic sinusoidal curvature. We show that the curvature can substantially change the band-structure in a manner that is reminiscent of the transition between a topologically trivial insulator to a non-trivial state. This is, however, not yet a true topological phase transition. In Section (IV), we demonstrate that for the Bernevig-Hughes-Zhang Hamiltonian^24^ describing a two-dimensional topological insulator, introducing a curvature does lead to a topological phase transition from a topologically trivial insulator to a non-trivial insulator with topological surface states.

## Physics of Curvature

We introduce the curvilinear coordinates *q*^*i*^, *i* = 1, 2, 3. Since our systems are two-dimensional, we need two independent coordinates to specify a point $\overrightarrow{r}$ on the surface. We denote these two coordinates as *q*^1^ and *q*^2^, so that a point on the surface is specified by $\overrightarrow{r}=(x({q}^{1},{q}^{2}),y({q}^{1},{q}^{2}),z({q}^{1},{q}^{2}))$. Two (not necessarily orthonormal) basis vectors tangential to the surface, *e*_*i*_, *i* = (1, 2), are ${e}_{i}\equiv {\partial }_{i}\overrightarrow{r}$. An arbitrary point in three-dimensional space $\overrightarrow{R}$ can then be specified in terms of the *q*^*i*^ s as $\overrightarrow{R}=\overrightarrow{r}({q}^{1},{q}^{2})+{q}^{3}\hat{n}({q}^{1},{q}^{2})$ where $\hat{n}$ ≡ *e*_1_ × *e*_2_/|*e*_1_ × *e*_2_| is the normal vector to the surface.

Let us first consider a generic two-dimensional system with spin *σ*^*j*^,

1 $$H=\frac{1}{2{m}_{0}}{p}^{i}{p}_{i}+{S}_{ij}{p}^{i}{\sigma }^{j}.$$

In the study of curvilinear systems, a central role is played by the tensor transformation rules

2 $${p}^{i}=\frac{\partial {q}^{i}}{\partial {\bar{q}}^{t}}{\bar{p}}^{t}$$

3 $${\sigma }^{i}=\frac{\partial {q}^{i}}{\partial {\bar{q}}^{t}}{\bar{\sigma }}^{t}$$

4 $${S}_{ij}=\frac{\partial {\bar{q}}^{k}}{\partial {q}^{i}}\frac{\partial {\bar{q}}^{t}}{\partial {q}^{j}}{\bar{S}}_{kt}$$

where the metric tensor matrix elements *g*_*ij*_ ≡ *e*_*i*_ ⋅ *e*_*j*_, and the momentum operator *p*^*i*^ acting to the right on a spatial wavefunction *ψ*, *p*^*i*^*ψ* ≡ −*ig*^*ij*^∂_*j*_*ψ*. These transformation rules allow us to, for instance, convert a system written in terms of the Cartesian coordinates (*x*, *y*, *z*) into the curvilinear *q*^*i*^ coordinates.

Our prescription for obtaining the Hamiltonian for our two-dimensional curved surface is as follows: We first start from the Hamiltonian for a *three*-dimensional system Eq. 1, and apply the tensor transformation rules Eqs 2 to 4 to rewrite the Hamitonian in terms of the curvilinear *q*^*i*^ coordinates. We then conceptually introduce a confining potential along the local surface normal direction, so that the particles are confined to *q*^3^ = 0, i.e. on the two-dimensional surface itself. This gives the effective Hamiltonian

5 $${\tilde{H}}_{KS}\psi =(-\frac{1}{2{m}_{0}}\frac{1}{\sqrt{g}}\frac{\partial }{\partial {q}^{m}}(\sqrt{g}{g}^{mn}\frac{\partial }{\partial {q}^{n}})-\frac{1}{8{m}_{0}}[\text{Tr}(\alpha {)}^{2}-4\mathrm{Det}(\alpha )]-i{S}_{im}{\sigma }^{i}{g}^{mu}\frac{\partial }{\partial {q}^{u}}+\frac{1}{2}i{S}_{i3}{\sigma }^{i}{\rm{Tr}}(\alpha ))\psi ,$$

where *m*_0_ is the rest mass of the electron, *g*(*q*^1^, *q*^2^) is the reduced metric tensor, and *α* is the Weingarten curvature matrix^26^. The indices *m*, *n* = (1, 2), and other index values range from 1 to 3. We emphasize that the confinement along the normal direction of the curved system will induce the curvature-controllable geometric potentials $-\frac{1}{8{m}_{0}}[{\rm{Tr}}{(\alpha )}^{2}-4{\rm{Det}}(\alpha )]$ and $\frac{1}{2}i{S}_{i3}{\sigma }^{i}{\rm{Tr}}(\alpha ))\psi$. The derivation of Eq. 5 is detailed in the Methods section.

For notational simplicity, let us introduce ${v}_{\mathrm{(2)}}\equiv \frac{1}{4}{\rm{Tr}}{({\alpha }_{m}^{n})}^{2}-4{\rm{Det}}({\alpha }_{m}^{n})$, ${v}_{\mathrm{(1)}}\equiv \frac{1}{2}{\rm{Tr}}({\alpha }_{m}^{n})$, and ${\partial }_{i}\equiv \frac{\partial }{\partial {q}^{i}}$, so that Eq. 5 can now be more concisely written as

6 $${\tilde{H}}_{KS}\psi =(-\frac{1}{2{m}_{0}}[{v}_{\mathrm{(2)}}+\frac{1}{\sqrt{g}}{\partial }_{m}(\sqrt{g}{g}^{mn}{\partial }_{n})]-i{S}_{im}{\sigma }^{i}{g}^{mu}{\partial }_{u}+i{v}_{\mathrm{(1)}}{S}_{i3}{\sigma }^{i})\psi .$$

## A 2D System with Sinusoidal Periodicity

**Figure 1:**
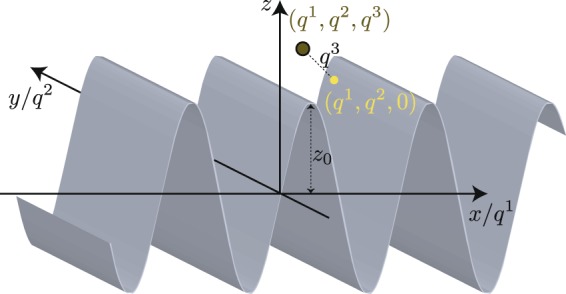
A schematic representation of the geometry considered for our numerical calculations consisting of a periodically curved surface described by (*x*, *y*, *z*) = (*q*^1^, *q*^2^, *z*_0_sin(2*q*^1^*π*/*L*)). A point lying on the surface is specified by two coordinates *q*^1^ and *q*^2^; an arbitrary point in three-dimensional space (red circle) can be specified by adding a third coordinate *q*^3^ which denotes the distance of the point away from the surface along the normal direction at its projection (green surface) onto the surface.

Now let us consider a periodically curved system, such as the one illustrated in Fig. 1 where the spin *σ* represents an orbital degree of freedom. We note in passing that a similar system has been studied in^27^ where the bulk of their results were obtained using a lattice Hamiltonian, whereas we study a continuum system here. By the Bloch theorem, an eigenstate of the system takes the form of $\psi (\overrightarrow{q})=\exp (i{k}_{i}{q}^{i})u(\overrightarrow{r})$ where *u* is a periodic function with the same periodicity as the system. Consider an eigenstate with energy *E*. Using the alternative form of the curvilinear Laplacian operator ${\nabla }^{2}=({\partial }_{i}+({\partial }_{i}\,\mathrm{ln}\,\sqrt{g})){g}^{ij}{\partial }_{j}={\nabla }_{i}({g}^{ij}{\partial }_{j})$ where ${\nabla }_{i}\equiv ({\partial }_{i}+({\partial }_{i}\,\mathrm{ln}\,\sqrt{g}))$ we have

7 $$\begin{array}{ll} & {\tilde{H}}_{KS}\psi =\psi E\\ \Rightarrow  & (-\frac{1}{2{m}_{0}}({\nabla }^{2}+{v}_{\mathrm{(2)}})-i({S}_{ij}{\sigma }^{i}{g}^{jl}{\partial }_{l}-{v}_{\mathrm{(1)}}{S}_{i3}{\sigma }^{i}))(u\,\exp (i\overrightarrow{k}\cdot \overrightarrow{r}))=(\exp (i\overrightarrow{k}\cdot \overrightarrow{r}))uE\\ \Rightarrow  & (-\frac{1}{2{m}_{0}}((({\nabla }_{i}+i{k}_{i}){g}^{ij}({\partial }_{j}+i{k}_{j}))+{v}_{\mathrm{(2)}})+({S}_{ij}{\sigma }^{i}{g}^{jl}({k}_{l}-i{\partial }_{l})+i{v}_{\mathrm{(1)}}{S}_{i3}{\sigma }^{i}))u=uE\\ \Rightarrow  & (\frac{1}{2{m}_{0}}(({p}_{i}+{k}_{i})({p}^{i}+{k}^{i})+{v}_{\mathrm{(2)}})+({S}_{ij}{\sigma }^{i}({p}^{j}+{k}^{j})+i{v}_{\mathrm{(1)}}{S}_{i3}{\sigma }^{i}))|u\rangle =|u\rangle E.\end{array}$$

We may therefore take the terms acting on *u* in the left hand side of Eq. 7 as an effective Hamiltonian, *H*_*u*_, for the periodic part of the Bloch state |*u*〉.

**Figure 2:**
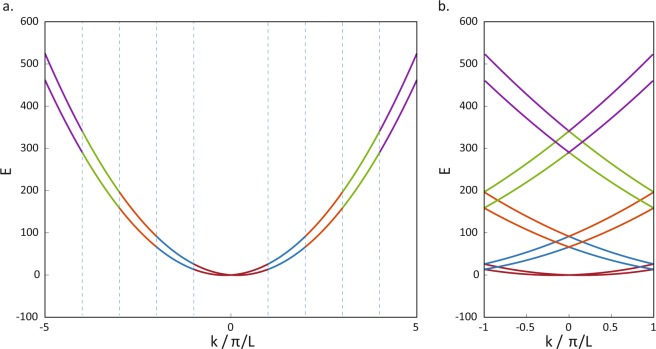
Panel (a) shows the plot of the dispersion relation *E* = *k*^2^/(2*m*) ± *βk* for an isotropic, translation-invariant system in the extended zone scheme. The dotted line denotes the Brillouin zone boundaries for an arbitrary choice of periodicity. Panel (b) shows the folding of the dispersion relations in panel (a) into the first Brillouin zone.

One consequence of having a periodic curvature is that the periodicity leads to the emergence of Brillouin zones, where the dispersion relations for all values of the crystal momentum $\overrightarrow{k}$ can be folded back into the first Brillouin zone. This folding emerges automatically in numerical calculations of the bandstructure involving the Bloch theorem, as we shall see. To familiarise the reader with the folding of the dispersion relation into the first Brillouin zone, let us first consider the dispersion of an isotropic, translation invariant system. Such a system can be considered to be a periodic system with an arbitrary period. Consider for example a dispersion relation of *E* = *k*^2^/(2*m*) ± *βk*. Figure 2 demonstrates how the dispersion relation is folded from the extended zone scheme, in which *k* extends to infinity, to the reduced zone scheme in which *k* is now limited to the first Brillouin zone. Multiple energy bands which exhibit both particle-like (positive ${\partial }_{k}^{2}E$) as well as hole-like (negative ${\partial }_{k}^{2}E$) dispersion relations now emerge.

For the numerical results which follow, we consider a sinusoidally periodic curved surface shown in Fig. 1 which, for simplicity, is taken to be translation invariant along the *q*^2^ direction. A point lying on the surface is given by (*x*, *y*, *z*) = (*q*^1^, *q*^2^, *z*_0_sin(2*πq*^1^/*L*)) where *L* is the spatial periodicity. Following the Bloch scheme described above, the translation invariance along the *q*^2^ direction leads to an eigenstate of the system of the form *ψ* = exp(*i*(*k*_1_*q*^1^ + *k*_2_*q*^2^))*u*(*q*^1^) where *u*(*q*^1^) has the same spatial periodicity as the curved surface. *u*(*q*^1^) can therefore be expanded in terms of a Fourier series

8 $$u({q}^{1})=\sum _{n}{c}_{n}\,\exp (2in\pi /L).$$

Substituting Eq. 8 into Eq. 7 gives

9 $$\begin{array}{c}\,\,\,\,(\frac{1}{2{m}_{0}}(({p}_{i}+{k}_{i})({p}^{i}+{k}^{i})+{v}_{\mathrm{(2)}})+(({S}_{ij}{\sigma }^{i}({p}^{j}+{k}^{j})+i{v}_{\mathrm{(1)}}{S}_{i3}{\sigma }^{i}))\sum _{n}{c}_{n}\,\exp \mathrm{(2}in\pi /L)=E\sum _{n}{c}_{n}\,\exp \mathrm{(2}in\pi /L)\\ \Rightarrow \frac{1}{L}\int {\rm{d}}{q}^{1}\sqrt{g}\,\exp (\,-\,2im\pi /L)(\frac{1}{2{m}_{0}}(({p}_{i}+{k}_{i})({p}^{i}+{k}^{i})+{v}_{\mathrm{(2)}})+({S}_{ij}{\sigma }^{i}({p}^{j}+{k}^{j})+i{v}_{\mathrm{(1)}}{S}_{i3}{\sigma }^{i}))\sum _{n}{c}_{n}\,\exp \mathrm{(2}in\pi /L)\\ \,\,\,\,=E(\frac{1}{L}\int {\rm{d}}{q}^{1}\sqrt{g}\,\exp (2i(n-m)\pi /)L)\end{array}$$

Note that in Eq. 9 the integration on both sides of the equal sign carries an additional factor of $\sqrt{g}$ due to the curvature of the system. This $\sqrt{g}$ factor is necessary in the definition of the matrix elements between two arbitrary states |Ψ〉 and |Φ〉, i.e. for an arbitrary Hermitian operator *O*, we have $\langle \Psi |O|{\rm{\Phi }}\rangle \equiv \int {\rm{d}}{q}^{1}\,\sqrt{g}{{\rm{\Psi }}}^{\ast }O{\rm{\Phi }}$, so that the Hermiticity condition 〈Ψ|*O*|Φ〉^*^ = 〈Φ|*O|*Ψ〉 is satisfied. In our numerical calculations, we expand Eq. 9 in terms of a sufficiently large number, *n*_max_, of exp(2*inπ*/*L*) basis modes sufficient to achieve convergence. Given the values of *k*_1_ and *k*_2_, we construct two 2*n*_max_ × 2*n*_max_ matrices which we denote as $\tilde{{\bf{H}}}$ and $\tilde{{\bf{A}}}$ from the left and right sides of the equal sign in Eq. 9 respectively by running the columns of the matrices over the *n* summation index and the rows over the *m* summation index. The additional factor of 2 in 2*n*_max_ comes from the spin degree of freedom. Eq. 9 then gives the generalized eigenvalue problem $\tilde{{\bf{H}}}{\rm{c}}=E\tilde{{\bf{A}}}{\rm{c}}$ which can be numerically solved to give the eigenvalues *E* and the eigenvectors **c**, which represent the column vectors of the *c*_*n*_ coefficients. (On a flat system where $\sqrt{g}=1$ everywhere, $\tilde{{\bf{A}}}$ reduces to an identity matrix.)

**Figure 3:**
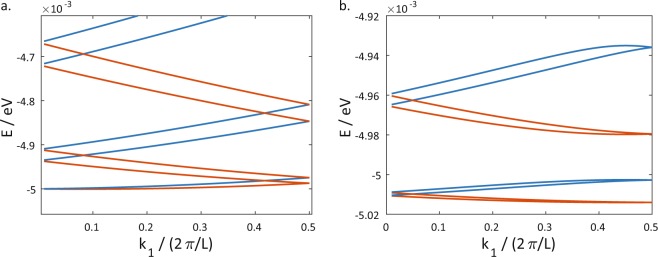
Panel (a) shows the dispersion relation of a *z*_0_ = 10 Å system, and panel (b) that of a *z*_0_ = 100 Å system with *α* = 0.1 eVÅ. The colours of the energy bands help to distinguish between how different bands and show how they evolve with changing *k*_1_ as the bands cross each other.

Figure 3 shows the dispersion relations for the lowest-energy bands at *k*_2_ = 0 for two SOI systems where the Hamiltonians for the three-dimensional systems take the form of $H={p}^{2}\mathrm{/(2}m)+\alpha \overrightarrow{p}\cdot \overrightarrow{\sigma }$, i.e. *S*_*ij*_ = *αg*_*ij*_. We used the numerical values *α* = 0.1 eVÅ, *m* = 6.94 × 10^−30^ kg and *L* = 500 Å in our calculations. We have shown only the dispersion relations for positive values of *k*_1_ as the dispersion relations are symmetric about *k*_1_ = 0.

The dispersion relation shows the two-fold Kramers’ degeneracy at the Time-Reversal Invariant Points (TRIMs) at *k*_1_ = 0 and *k*_1_ = *π*/*L*. The dispersion relation for the less curved system in panel (a) resembles that of the flat system in Fig. 2b where a particle-like band with a positive $({\rm{sign}}({k}_{1}){\partial }_{{k}_{1}}E)$ is degenerate with a hole-like band with a negative $({\rm{sign}}({k}_{1}){\partial }_{{k}_{1}}E)$ at the TRIMs at *k*_1_ = 0, ±*π*/*L*. Increased curvature leads to a scenario similar to that in panel (b) where the bands now ‘switch partners’ and particle-like (hole-like) bands pair with particle-like (hole-like) bands at the TRIMs. Band gaps, i.e. energy ranges without propagating states, now emerge at *k*_2_ = 0.

While the partner switching and band gap opening are somewhat reminiscent of a topological phase transition in topological insulators, the Hamiltonian of Eq. 1 does not yet support topological phase transitions. This can be seen from the fact that the opening of the band gaps in panel (b) of Fig. 3 does not result in the emergence of topological edge states when an edge is introduced into the system.

## Curvature-Controllable Quantum Phase Transitions with SOC

We will now turn our attention to a Hamiltonian in which a topological phase transition can be induced by the curvature. A topological insulator (TI) is a new state of quantum matter with an insulating bulk and a topologically protected conducting surface^24,25,28–31^. Bernevig, Hughes and Zhang (BHZ) introduced the Hamiltonian

10 $$H=-\,D{k}_{\parallel }^{2}{{\bf{I}}}_{4}+(M-B{k}_{\parallel }^{2}){\sigma }_{z}{{\bf{I}}}_{s}+A({\overrightarrow{k}}_{\parallel }\cdot \overrightarrow{\sigma }){s}_{z}$$

to describe a quantum-well (QW) system on the *xy* plane hosting a two-dimensional topological insulator state^24^. Here, the *σ* s are Pauli matrices referring to an orbital degree of freedom, the *s*_*z*_ is the *z* Pauli matrix referring to the real spin degree of freedom, *k*_∥_ is a vector on the *xy* plane, and *A*, *B*, *M* and *D* are quantum-well dependent parameters. The term containing *s*_*z*_ represents a spin orbit coupling term. (We have dropped a term corresponding to a shift of the energy origin). Notice that in this Hamiltonian the two real spin orientations are decoupled to each other, so we may consider just a single real spin and get the corresponding results for the other spin by applying time reversal. We thus consider the real spin up. For our numerical calculations, we use the numerical values of *A* = −5 eVÅ^2^, *B* = −20 eVÅ^2^ and *D* = 0.5 eVÅ^2^ which are rounded values approximately equal in magnitude to those for the HgTe quantum well system in the original BHZ paper.

**Figure 4:**
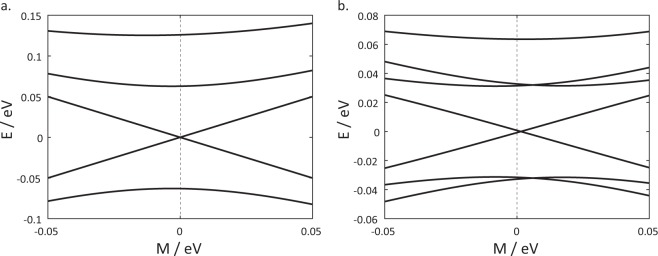
Panel (a) shows the variation of the energy of the energy bands at *k* = 0 on a flat QW with the variation of *M*. Panel (b) shows the variation of the energies of the energy bands at *k* = 0 on a curved QW.

With *A*, *B* and *D* fixed, a phase transition occurs when the value of *M* changes sign. Panel (a) of Fig. 4 shows the variation with the parameter *M* of the energies of the energy bands at *k* = 0 of a flat QW. (More than two bands appear in the figure because of the folding of the energy bands into the first Brillouin zone.) The two bands just above and below *E* = 0 are the conduction and valence bands respectively. As *M* is gradually increased from a large negative value, the energy separation between the two bands shrinks until it reaches zero. The bands then cross each other as *M* is increased further. The gap closing and subsequent band crossing at *M* = 0 corresponds to a quantum phase transition from a topologically non-trivial state to a trivial state.

We now consider the corresponding case for a curved QW system. In order to describe a *curved* surface, let us first extend Eq. 10 to three dimensions with the replacement of *k*_∥_ with $-{\nabla }_{\mathrm{(3)}}^{2}$ where the subscript (3) indicates that this is the Laplacian operator in three dimensions. We then confine the resulting Hamiltonian to our desired curved two-dimensional surface similar to what we did in the previous section. This gives

11 $$\begin{array}{ccc}H\psi  & = & (D({\nabla }_{\mathrm{(2)}}^{2}+{v}_{\mathrm{(2)}})){{\bf{I}}}_{\sigma }+(M+B({\nabla }_{\mathrm{(2)}}^{2}+{v}_{\mathrm{(2)}}){\sigma }_{z}\\  &  & +A(-i{\sigma }^{i}{\partial }_{i}+i{v}_{\mathrm{(1)}}{\sigma }^{3}))\psi \end{array}$$

where *v*_(1)_ and *v*_(2)_ are as defined in the text before Eq. 5.

The introduction of a curvature can shift the value of *M* at which the phase transition occurs. Panel (b) of Fig. 4 shows that in a curved structure the band crossing point is shifted away from *M* = 0 towards a small positive value of *M*. This shift can be intuitively understood in the following manner: In the Hamiltonian of a flat system Eq. 10, *M* is the momentum-independent term which appears together with *σ*_*z*_. In the Hamiltonian for the curved system Eq. 11, there is an additional momentum-independent term *Bv*_(2)_*σ*_*z*_ which is associated with *σ*_*z*_. The phase transition is therefore controlled by the sign of (*M* + *Bv*_(2)_) rather than that of *M* alone. For curved surfaces of the form (*x*, *y*, *z*) = (*q*^1^, *q*^2^, *z*(*q*^1^)), *v*_(2)_ is always negative. This accounts for the small positive value of *M* at the transition point.

**Figure 5:**
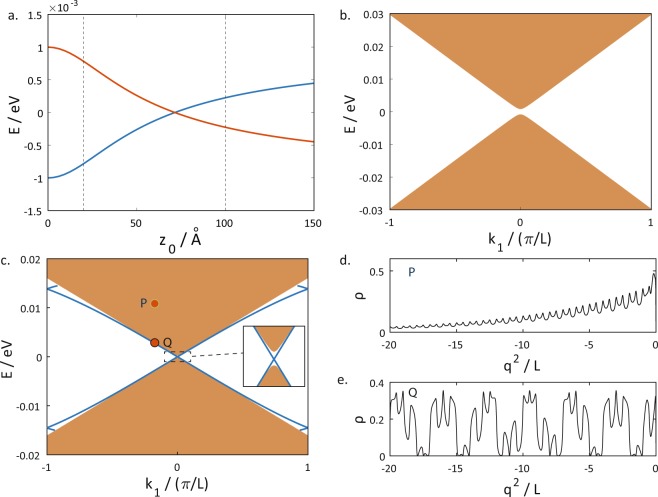
(**a**) The energies of the conduction and valence bands as a function of the sinusoidal amplitude *z*_0_ for a fixed value of *M* = 0.001 eV. Panels (b) and (c) show the dispersion relations when an interface to the vacuum parallel to the *e*_1_ direction is introduced at b. *z*_0_ = 20 Å, and c. *z*_0_ = 100 Å (both values of *z*_0_ are indicated by the dotted lines on panel (a)). Surface states localized near the edges, represented by the thick blue lines, are present on the curved surface and absent in the less curved one. The continuum of bulk states are represented by the solid green areas. The inset of panel (c) is a zoom in of the boxed area and shows the bulk band gap crossed by the topological surface states. Panels (d) and (e) show the spatial density distribution of two exemplary surface and bulk states indicated by the two dots P and Q in panel (b).

To gain another perspective on the controllability of the phase transition by the curvature, we now fix the value of *M* and vary *z*_0_, the amplitude of the sinusoidal variation instead. Panel (a) of Fig. 5 shows that as the curvature is increased from 0, the energy separation between the conduction and valence bands shrinks until the bands cross each other at a critical value of curvature, and a band inversion occurs. This band inversion is associated with a topological phase transition. When an interface to a topologically trivial material is introduced, here by imposing a hard wall potential at *q*^2^ = 0 and making the system a semi-infinite one extending from *q*^2^ = −∞ to *q*^2^ = 0, surface states localized near the interface emerge only on one side of band inversion (panel (c)) and not the other (panel (b)). A detailed account of how the surface states are obtained is provided in the Methods section. The fact that the states which emerge in the bulk band gap are surface states for the more curved structures can be ascertained by verifying that the density distribution of these topological surface states decay exponentially away from the surface (panel (d)) unlike the topologically trivial bulk states (panel (e)) which show a standing wave pattern.

## Conclusion

In this work, we first showed the derivation of the Hamiltonian for a generic curved nanostructure. We next introduced a numerical scheme to calculate the bandstructure of a a periodically curved nanostructure. Using the Hamiltonian we derived, we showed that introducing curvature into a two-dimensional system can open up band gaps and lead to partner switching at the TRIMs. We then studied the BHZ Hamiltonian for a two-dimensional quantum well. We demonstrated that a quantum phase transition from a topologically trivial phase to a non-trivial phase can be induced by introducing a curvature.

## Methods

### Dimensional reduction

Let us consider an arbitrary operator *O* in a three-dimensional system in which we do not confine the system to lie on the two-dimensional system yet, and denote the determinant of the three-dimensional metric tensor with an upper case *G*, and the determinant of the two-dimensional surface as a lower case *g*. We denote the three-dimensonal wavefunction ${\mathscr{X}}$(*q*^1^, *q*^2^, *q*^3^) = ${\mathscr{X}}$_*t*_(*q*^1^, *q*^2^)${\mathscr{X}}$_*n*_(*q*^3^). Consider the expectation value of *O* over an infinitesimal volume element

12 $$\begin{array}{rcl}{{\mathscr{X}}}^{\ast }O{\mathscr{X}}\,{\rm{d}}V & = & {{\mathscr{X}}}^{\ast }O{\mathscr{X}}\sqrt{G}{\rm{d}}{q}^{1}{\rm{d}}{q}^{2}{\rm{d}}{q}^{3}\\  & = & {{\mathscr{X}}}_{n}^{\ast }[{\{{(\frac{G}{g})}^{\frac{1}{4}}{\chi }_{t}\}}^{\ast }O\{{(\frac{G}{g})}^{\frac{1}{4}}{{\mathscr{X}}}_{t}\}\,\sqrt{g}{\rm{d}}{q}^{1}{\rm{d}}{q}^{2}]{\chi }_{n}{\rm{d}}{q}^{3}\\  & = & {\chi }_{n}^{\ast }[(\sqrt{f}{\chi }_{t}^{\ast })O(\sqrt{f}{\chi }_{t})\,{\rm{d}}S]{\chi }_{n}{\rm{d}}{q}^{3}\end{array}$$

This suggests that we should identify the effective wavefunction on the two-dimensional surface, *ψ*, as $\psi =\sqrt{f}{{\mathscr{X}}}_{t},f\equiv {(\frac{G}{g})}^{\mathrm{1/2}}$ as the effective wavefunction on the two-dimensional surface. Consequently, we have ${{\mathscr{X}}}_{t}=\sqrt{\frac{1}{f}}\psi$ so that substituting this into the *p*^*i*^*p*_*i*_ operator gives

13 $$\begin{array}{ll} & -{\nabla }_{\mathrm{(3)}}^{2}{{\mathscr{X}}}_{t}\\ = & -({\tilde{\nabla }}_{\mathrm{(2)}}^{2}{\mathscr{X}}+\frac{1}{G}{\partial }_{3}(\sqrt{G}{\partial }_{3}{\mathscr{X}}))\\ = & -({\tilde{\nabla }}_{\mathrm{(2)}}^{2}({f}^{-\frac{1}{2}}\psi )+\frac{1}{4{f}^{\frac{3}{2}}}(({({\partial }_{3}f)}^{2}-2f{\partial }_{3}^{2}\,f)\psi +4{f}^{2}{\partial }_{3}^{2}\psi ))\end{array}$$

where ${\tilde{\nabla }}_{\mathrm{(2)}}^{2}\equiv {\sum }_{i,j}^{2}\frac{1}{\sqrt{G}}{\partial }_{i}(\sqrt{G}{G}^{ij}{\partial }_{j})$. This differs from the non-tilde version of ${\nabla }_{\mathrm{(2)}}^{2}$ in Eq. 11 in that the metric tensors and metric tensor matrix elements that appear in the ${\tilde{\nabla }}_{\mathrm{(2)}}^{2}$ are the *three*-dimensional metric tensors, which have *q*^3^ dependence, while ${\nabla }_{\mathrm{(2)}}^{2}$ is the Laplacian operator on the two-dimensional surface where *q*^3^ is fixed to 0. We take it as a given that

14 $$f=1+{\rm{Tr}}(\alpha ){q}^{3}+{\rm{Det}}(\alpha )({q}^{3}{)}^{2}.$$

Setting *q*^3^ = 0 so that we are only considering states confined to our surface and dropping the *q*^3^ derivatives of *ψ* gives

15 $$\begin{array}{ll} & -{\nabla }_{\mathrm{(3)}}^{2}{{\mathscr{X}}}_{t}{|}_{{q}^{3}\to \mathrm{0,}{\partial }_{3}\psi \to \mathrm{0,}{\partial }_{3}^{2}\psi \to 0}\\ = & -({\nabla }_{\mathrm{(2)}}^{2}\psi +\frac{1}{4}(({\rm{Tr}}(\alpha {))}^{2}-4{\rm{Det}}(\alpha ))\psi ).\end{array}$$

Similarly, we have

16 $$-i{\partial }_{3}{{\mathscr{X}}}_{t}{|}_{{q}^{3}\to \mathrm{0,}{\partial }_{3}\psi \to \mathrm{0,}{\partial }_{3}^{2}\psi \to 0}=\frac{i}{2}{\rm{Tr}}(\alpha )\psi .$$

### Surface states

In our numerical calculations we conceptually truncate the system at *q*^2^ = 0 so that instead of extending from *q*^2^ = −∞ to *q*^2^ = ∞ the surface now extends from *q*^2^ = −∞ to *q*^2^ = 0. The wavevector *k*_1_ is still a good quantum number. An eigenstate of the *semi*-infinite system for a given value of *k*^1^ and energy *E*, if it exists, can be written as a linear combination of the *infinite* length eigenstates subjected to the boundary condition that the wavefunction vanishes at *q*^2^ = 0.

Let us therefore describe how we numerically obtain the infinite-length eigenstates when the energy *E* and *k*^1^ are given. Using a similar scheme to that outlined in the discussion surrounding Eq. 9, we construct a 2*n*_max_ × 2*n*_max_ numerical matrix representation of the Hamiltonian Eq. 11, **H**. We have

17 $${\bf{Hc}}=E{\bf{Ac}}$$

18 $$\Rightarrow ({{\bf{H}}}_{\mathrm{(0)}}+{{\bf{H}}}_{\mathrm{(1)}}{k}_{2}+{{\bf{H}}}_{\mathrm{(2)}}{({k}_{2})}^{2}){\bf{c}}=E{\bf{Ac}}$$

19 $$\Rightarrow (\begin{array}{cc}{{\bf{H}}}_{\mathrm{(0)}}-E{\bf{A}} & {{\bf{H}}}_{\mathrm{(1)}}\\ {\bf{0}} & {\bf{I}}\end{array})(\begin{array}{c}{\bf{c}}\\ {k}_{2}{\bf{c}}\end{array})={k}_{2}(\begin{array}{cc}{\bf{0}} & -{{\bf{H}}}_{\mathrm{(2)}}\\ {\bf{I}} & {\bf{0}}\end{array})(\begin{array}{c}{\bf{c}}\\ {k}_{2}{\bf{c}}\end{array})$$

In going from Eq. 17 to Eq. 18 we decomposed **H** into a part independent of *k*_2_, **H**_(0)_, a part linear in *k*_2_, **H**_(1)_*k*_2_ and a part quadratic in *k*_2_, **H**_(2)_(*k*_2_)^2^. Eq. 19 is a rewriting of Eq. 18 into a generalized eigenvalue problem where the eigenvalues are the *k*_2_ s and the eigenvectors (***c***,*k*_2_***c***)^T^. Since the block matrices in Eq. 19 are 4*n*_max_ × 4*n*_max_ matrices, there are 4*n*_max_ eigenvectors and corresponding values of *k*_2_. The *k*_2_ s may, in general, be complex.

One consequence of introducing the boundary is that evanescent states with complex *q*^2^ wavevectors may now be admissible in the system if the imaginary parts of the wavevectors are negative, so that these states decay away exponentially away from the surface. (Evanescent states cannot exist in the infinitely long system since they will blow up exponentially at either *q*^2^ = ∞ or *q*^2^ = −∞). The allowed states in our semi-infinite systems are therefore those with real values of *k*_2_, and those where the imaginary parts of *k*_2_ are negative. Depending on the given values of *E* and *k*_1_, the number of allowed states may range from 2*n*_nmax_, where all the *k*_2_ values have imaginary parts, to 4*n*_nmax_. On the other hand, the boundary condition that the wavefunction vanishes at *q*^2^ = 0 imposes 2*n*_nmax_ constraints. It is always possible to find a non-trivial linear combination of vectors which sum up to zero when the number of vectors to choose from is more than the dimensionality of the vectors. Eigenstates for the *semi-infinite* system therefore exist when the the number of allowed *infinite*-length eigenstates exceeds 2*n*_nmax_.

For the case when the number of allowed infinite-length eigenstates is exactly 2*n*_nmax_, we construct a 2*n*_max_ × 2*n*_max_ matrix **V** whose columns consist of the allowed eigenstates. A linear combination of eigenstates which satisfies the boundary condition exists if and only if the determinant of **V** is zero.
